# The Protective Role of Prenatal Alpha Lipoic Acid Supplementation against Pancreatic Oxidative Damage in Offspring of Valproic Acid-Treated Rats: Histological and Molecular Study

**DOI:** 10.3390/biology9090239

**Published:** 2020-08-20

**Authors:** Fatma M. Ghoneim, Hani Alrefai, Ayman Z. Elsamanoudy, Salwa M. Abo El-khair, Hanaa A. Khalaf

**Affiliations:** 1 Histology and Cell Biology Department, Faculty of Medicine, Mansoura University, Mansoura 35516, Egypt; fatmaghoneim@mans.edu.eg (F.M.G.); hanaaattia@mans.edu.eg (H.A.K.); 2 Medical Biochemistry and Molecular Biology Department, Faculty of Medicine, Mansoura University, Mansoura 35516, Egypt; aymanzs@mans.edu.eg (A.Z.E.); salwakhair@mans.edu.eg (S.M.A.E.-k.); 3 Department of Internal Medicine, Infectious Diseases Div., College of Medicine, University of Cincinnati, Cincinnati, OH 45267, USA; 4 Clinical Biochemistry Department, Faculty of Medicine, King Abdulaziz University, Jeddah 21465, Saudi Arabia

**Keywords:** valproic acid, alpha lipoic acid, pancreas, nrf2, bcl-2, caspase-3, oxidative damage, antioxidants

## Abstract

Background: Sodium valproate (VPA) is an antiepileptic drug (AED) licensed for epilepsy and used during pregnancy in various indications. Alpha-lipoic acid (ALA) is a natural compound inducing endogenous antioxidant production. Our study aimed to investigate the effect of prenatal administration of VPA on the pancreas of rat offspring and assess the potential protective role of ALA co-administration during pregnancy. Methods: Twenty-eight pregnant female albino rats were divided into four groups: group I (negative control), group II (positive control, ALA treated), group III (VPA-treated), and group IV (VPA-ALA-treated). The pancreases of the rat offspring were removed at the fourth week postpartum and prepared for histological, immune-histochemical, morphometric, molecular, and oxidative stress marker studies. Results: In group III, there were pyknotic nuclei, vacuolated cytoplasm with ballooning of acinar, α, and β cells of the pancreas. Ultrastructural degeneration of cytoplasmic organelles was detected. Additionally, there was a significant increase in oxidative stress, a decrease in insulin-positive cell percentage, and an increase in glucagon positive cells in comparison to control groups. Moreover, VPA increased the gene expression of an apoptotic marker, caspase-3, with a decrease in anti-apoptotic Bcl2 and nuclear factor erythroid 2-related factor 2 (Nrf2) transcriptional factor. Conversely, ALA improved oxidative stress and apoptosis in group VI, and a consequent improvement of the histological and ultrastructure picture was detected. Conclusion: ALA co-administration with VPA significantly improved the oxidative stress condition, histological and morphometric picture of the pancreas, and restored normal expression of related genes, including Nrf2, caspase-3, and Bcl-2. Administration of α-lipoic acid has a protective effect against VPA-induced pancreatic oxidative damage via its cytoprotective antioxidant effect.

## 1. Introduction

Antiepileptic drugs (AEDs) are commonly used during pregnancy and are prescribed in various drug medication [1,2]. Sodium valproate (VPA) is a drug medication that was previously licensed for epilepsy and bipolar psychiatric disorders, as well as migraine prophylaxis [3,4]. Prenatal administration of AED could be hazardous for the fetus because these drugs cross the placenta and may be associated with adverse outcomes [5]. It is reported that valproate constitutes about 25% of antiepileptic drugs prescribed in pregnancy despite its possible teratogenic effect [3,6].

Prenatal exposure of embryos to valproic acid, especially during the period of organogenesis, can lead to abnormalities in the structure of the pancreas and, consequently, its function. These abnormalities occur due to defects in proliferation and differentiation in pancreatic cells during embryonic development [7,8]. Moreover, VPA is associated with the induction of oxidative stress, inflammatory reactions including pancreatitis, and apoptosis [9,10,11].

Alpha-lipoic acid (ALA) is a naturally occurring dithiol compound. It is synthesized from octanoic acid in the mitochondria. ALA has two forms: an oxidized form (lipoic acid) and reduced form (dihydrolipoic acid, DHLA). DHLA acts as a potent antioxidant, and it is used as a readily available dietary supplement. ALA improves endothelial function in patients with type-2 diabetes, Alzheimer’s disease, and other conditions [12].

Besides the role of α-lipoic acid as a potent antioxidant, it also acts as an inducer of endogenous antioxidant production. Moreover, it has a protective effect on hepatic and pancreatic injury [13]. It is safe to be administered during pregnancy, and no adverse effect was reported in mothers or newborns [14].

The purpose of this study is to investigate, at ultrastructure, biochemical, and molecular levels, the effect of prenatal administration of valproic acid on the pancreas of rat offspring, and to assess the potential protective role of ALA co-administration during pregnancy.

## 2. Materials and Methods

### 2.1. Chemicals

- Sodium valproate (VPA): in the form of Depakine ampoule (4 mL) in a concentration of 100 mg/mL, it was purchased from Sanofi Aventis France Company (Paris, France).
- Alpha-lipoic acid (ALA): purchased from Sigma-Aldrich (T5625: Sigma-Aldrich, St. Louis, MO, USA) in the form of a faint yellow powder, 25 g.

### 2.2. Experimental Protocol

The experimental protocol of the study was approved by the Institutional Review Board (IRB) of the Faculty of Medicine, Mansoura University (code number: R.19.12.695.R1.R2). Animals were used per the Animal Welfare Act, and Guide for Care Use approved by Mansoura Experimental Research Center. Female Sprague Dawley albino rats weighing 200–250 g were used. They were matched with male rats overnight. Copulation was confirmed by plug control examination every morning. The presence of a plug in the vagina was considered as day zero of gestation. After evident pregnancy, a total of 28 pregnant females were housed in metal cages with meshes. They were maintained with free access to commercial food consisting of standard laboratory rat chow and had free access to drinking water. The pregnant rats were allocated equally into four groups, as follows.

Group I (negative control group): included seven rats received an intraperitoneal injection of saline. Group II (positive control group): included seven rats received ALA by intraperitoneal injection at a dose of 30 mg/kg body weight in Tris buffer (pH = 7.4) between gestational days 0 and 15 [15]. Group III (VPA-treated group): included seven pregnant rats; received valproic acid at a single dose of 600 mg/kg (6 mL/kg body weight) by intraperitoneal injection on day 13 of gestation [16]. Group IV (VPA- and ALA-treated group): included seven pregnant rats; treated with VPA as in group III and received ALA through intraperitoneal injection at a dose of 30 mg/kg body weight between gestational days 0 and 15.

### 2.3. Sampling and Preparation of the Specimen

The experiment started with a total number of 63 pups, 28 pups died during the course of the experiment, 35 pups completed, so the pup mortality rate in the model was 44% (28 out of 63) and finally, we selected 28 pups for the statistical analysis. At the age of 4 weeks postpartum, seven pups were selected from each experimental group. After 12 h overnight fasting, each rat was anesthetized by ether, and a three milliliters blood sample was collected under the aseptic condition from retro-orbital venous plexus using a disposable plastic syringe. Blood samples were withdrawn and used for serum preparation, stored at −80 °C for biochemical analysis of catalase activity, reduced glutathione (GSH) and superoxide dismutase (SOD). All pups of experimental rats from each group (total 28) were sacrificed under diethyl ether anesthesia, and the pancreas was removed for histological, immunohistochemical, biochemical, and molecular studies.

The pancreas was excised, weighed, divided, and immediately frozen in liquid nitrogen or fixed in 10% buffered formalin. Paraffin sections (4–5 μm thick) were prepared and stained with H&E stains (the first specimen). The second pancreatic tissue specimen was prepared for electron microscopic examination. The liquid nitrogen frozen pancreatic tissue sample (the third specimen, 48 mg in weight) was used for total RNA extraction and real-time qPCR analysis of expression of Nrf2, caspase-3, and Bcl-2. Tissue homogenate for biochemical investigation was also prepared for MDA and total antioxidant activity (TAC).

### 2.4. Histological Study

The histological study of our work started by the fixation of pancreatic specimens from experimental rat offspring in Bouin solution followed by dehydration in ascending series of alcohol, clearing in two changes of xylene and embedding in paraffin. After embedding, 5–6 µm thickness sections were obtained, mounted on clean slides, and stained with hematoxylin and eosin (H&E) according to Bancroft and Layton [17].

### 2.5. Electron Microscopic Study

The pancreas was cut into small parts and fixed in 2.5% glutaraldehyde with Sodium Cacodylat buffer (0.1 M, pH 7.2) for about 2 h. After that, the samples were washed using the same buffer, and then post-fixation was done by using osmium tetroxide with phosphate-buffer (1%) for 2 h at room temperature. The samples were dehydrated in an increasing concentration of ethanol then immersed in propylene oxide. Semithin sections were stained with toluidine blue and examined by light microscope. Then the samples were sectioned (at 80–90 nm) and stained with uranyl acetate and lead citrate [18]. The examination was done with a transmission electron microscope (JEOL TEM CS 100) in the Electron Microscopic Unit, Faculty of Science, El-Shatby, Alexandria University, Alexandria, Egypt.

### 2.6. Immunohistochemical (IHC) Study

Immunohistochemical procedures for insulin and glucagon were carried out by using the streptavidin-biotin-peroxidase staining technique [19]. Sections were deparaffinized, rehydrated via decreasing concentrations of alcohol. To quench the endogenous peroxidase and the non-specific binding spots for antibodies, the sections were washed with hydrogen peroxide (0.3%) for 20 min and normal bovine serum (1:5 diluted TRIS) for another 20 min at room temperature, respectively. Slides were washed in phosphate-buffered saline and put in 10% normal goat serum for 30 min to reduce non-specific binding. Then incubation of the specimen was done for 1 h with anti-sera containing primary antibodies for insulin antibody (Pig polyclonal antibody, A0564; Dako, Carpentaria, CA, USA, 1:100) (No. P01315, Q9TSJ5) and for glucagon antibody (cat #PA5-13442) (rabbit polyclonal antibody, supplied by Thermo Fisher Scientific, Cramlington, UK, 1:50). Then incubated again with biotinylated secondary antibody (Dako-K0690; Dako Universal LSAB Kit, 1:100) and streptavidin horseradish peroxidase (Dako-K0690) for 30 min, and then 3,3′-diaminobenzidine tetrahydrochloride (Sigma-D5905; Sigma-Aldrich Company Ltd., Gillingham, UK) substrate kit for 10 min for immunolabelling. Lastly, the sections were counterstained by hematoxylin, dehydrated, cleared, and mounted in DPX. Negative control sections were prepared in the same way after omitting the primary antibody.

For morphometric analysis of insulin and glucagon immunoreactivity, all of the immunohistochemically stained sections were examined for the insulin and glucagon hormones. For calculation of the percentage of the positive cells of each hormone in each islet, all islets (in at least five high-power fields) were selected, and the whole number of nuclei in each of them was calculated. The average percentage of the positive cells for each hormone was obtained. This morphometric assessment was done via the Database Manual Cell Sens Life Science Imaging Software System (Olympus Corporation, Tokyo, Japan).

### 2.7. Biochemical Analysis

Estimation of pancreatic tissue content of malondialdehyde (MDA) was done by using the thiobarbituric acid reaction explained by Draper et al., and the result was stated in nmol/g tissue [20]. Total antioxidants were analyzed according to the method described by Koracevic et al. via the kit (Cat. No. # TA 25 12) delivered from Bio-Diagnostics, Dokki, Giza, Egypt. The result was presented as mmol/L [21].

Catalase activity (Catalase Activity Assay Kit-ab83464, Abcam, Hong Kong, China) reduced glutathione (Reduced Glutathione (GSH), Endogenous antioxidant ab142044, Abcam) and Superoxide Dismutase (SOD) (Superoxide Dismutase Activity Assay Colorimetric Kit-ab65354, Abcam) were determined according to the manufacturer’s instruction in serum samples [22,23,24]. The parameters were represented as U/mL, μmol/mL, and U/mL, respectively.

### 2.8. Molecular Study

Real-Time qPCR (RT-qPCR): Total RNA from rat pancreatic tissue samples (≈25 mg) was extracted using Trizol Reagent (Invitrogen) according to the manufacturer instructions. Reverse transcription reaction for cDNA synthesis was performed with ≈250 ng total RNA using Maxima First Strand cDNA Synthesis Kit (Thermo Scientific, Waltham, MA, USA, cat No. #K1641). The rat pancreatic mRNA expression of Nrf2, caspase-3, and Bcl-2 were quantified by real-time PCR on the Applied Biosystem 7500, real-time PCR detection system (Life Technology, Carlsbad, CA, USA) with Applied Biosystem SYBR^®^ Green PCR Master Mix (2X) (Life Technology, USA, cat. No. 4344463). Reaction mixtures were incubated for 10 min at 95 °C, followed by 40 cycles of 15 s at 95 °C, 1 min at 60 °C and, finally, 15 s at 95 °C, 1 min at 60 °C, and 15 s at 95 °C. The primer sequences used were rat Nrf2: forward, 5′-CAC ATC CAG ACA GAC ACC AGT-3′; reverse, 5′-CTA CAA ATG GGA ATG TCT CTG C-3′ [25], rat caspase-3: forward, 5′-GTG GAA CTG ACG ATG ATA TGG C-3′; reverse, 5′-CGC AAA GTG ACT GGA TGA ACC-3′, rat Bcl-2: forward, 5′-TGT GGA TGA CTG ACT ACC TGA ACC-3′; reverse 5′-CAG CCA GGA GAA ATC AAA CAG AGG-3′. The primers sequences for rat β-actin were 5′-AAG ATC CTG ACC GAG CGT GG-3′ (Forward) and 5′-CAG CAC TGT GTT GGC ATA GAG G-3′ (Reverse) [26]. The expression of the analyzed genes was normalized to that of the internal control gene, the β-actin, using the comparative ΔΔCT method.

### 2.9. Statistical Analysis

All the obtained data were presented as mean value ± SD. Comparisons were made via analysis of variance followed by Tukey’s test, with SPSS for Windows (15.0 Version). Changes were considered statistically significant when *p* < 0.05 and *p* < 0.01 highly significant.

## 3. Results

### 3.1. Histological Results

#### 3.1.1. Light Microscopic Results

**Figure 1:**
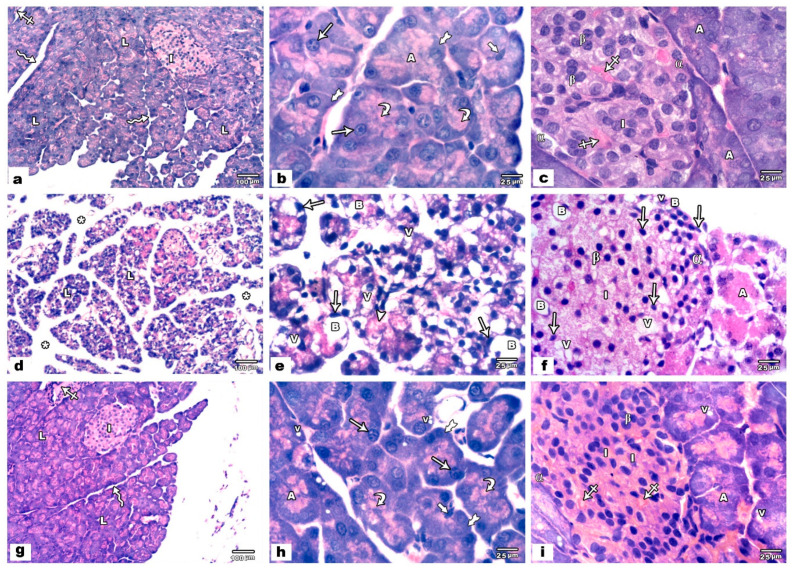
Photomicrographs of H&E-stained pancreatic sections. Control sections (**a**–**c**). (**a**) The pancreas of these groups consists of several adjacent lobules (L) of variable sizes and shapes separated by thin septa (zigzag arrows) containing blood vessels (crossed arrow). Pale pink oval islets of Langerhans (I) are also seen. (**b**) The lobules exhibit tightly packed acini (A) with narrow lumina occupied by small centroacinar cells with oval nuclei (thick arrow). The cells of the acini are pyramidal in shape with rounded, basal, and vesicular nuclei (arrow). The cytoplasm of them shows basal basophilia (tailed arrow) and apical acidophilic zymogen granules (curved arrow). (**c**) The islets of Langerhans (I) are present among the pancreatic acini (A) and formed of β cells (β) in the center of the islets and peripheral α cells (α) with pale cytoplasm and rounded vesicular nuclei disconnected by blood capillaries (crossed arrow). Sodium valproate (VPA) treated pancreatic sections (**d**–**f**). (**d**) Widening of the interstitial tissue (asterisk) between the lobules (L) is noticed. (**e**) Many acinar cells show pyknotic nuclei (arrows), vacuolated cytoplasm (V), and ballooning of some cells (B). Acidophilic fibrillar cytoplasm is seen in some acinar cells (arrow head). (**f**) The islets of Langerhans (I) are present among the pancreatic acini (A) and show pyknotic nuclei (arrows) with ballooning (B) and vacuolation (V) of the cytoplasm of α (α) and β (β) cells. VPA and alpha-lipoic acid (ALA) treated pancreatic sections (**g**–**i**). (**g**) The pancreas of this group consists of several adjacent lobules (L) separated by thin septa (zigzag arrows) containing blood vessels (crossed arrow) and islets of Langerhans (I) (**h**) The lobules exhibit many closely packed acini (A) with narrow lumina. The lumen of the acini is occupied by centroacinar cells with oval nuclei (thick arrow). The cells of the acini are pyramidal in shape with rounded, basal, vesicular nuclei (arrow) its cytoplasm has basal basophilia (tailed arrow) and apical acidophilic zymogen granules (curved arrow). Vacuolated cytoplasm (V) is seen in some acinar cells. (**i**) The islets of Langerhans (I) are present in between the pancreatic acini (A) and formed of β cells in the center of the islets (β) and peripheral α cells (α) disconnected by blood capillaries (crossed arrow).

Examination of H&E stained sections of the pancreas of the offspring of control groups (groups I and II) revealed the normal histological exocrine and endocrine compartments of the pancreas. The exocrine compartment was formed of several adjacent lobules of variable sizes and shapes separated by thin, delicate fibrous connective tissue septa containing blood vessels. The endocrine compartment (the islets of Langerhans) was observed among the exocrine one. (Figure 1a). The lobules of the exocrine compartment exhibited many acini with narrow lumina occupied by small centroacinar cells with oval nuclei. The acini were tightly packed and lined by pyramidal cells with basal, rounded open face nuclei, and the cytoplasm appeared with basal basophilia and apical acidophilic zymogen granules (Figure 1b). Islets of Langerhans were present among the acinar cells as pale pink oval or rounded areas with no apparent capsule between them. It was formed of clusters of cells (peripheral α cells and central β cells) with pale cytoplasm and rounded vesicular nuclei. These cells of islets were disconnected by blood capillaries (Figure 1c).

In the pancreas of offspring of VPA exposed rats, hematoxylin and eosin-stained sections revealed striking changes of both exocrine and endocrine compartments compared with that of control groups. There was a widening of the interstitial tissue between the lobules (Figure 1d). Many acinar cells showed pyknotic nuclei, vacuolated cytoplasm with ballooning of some cells. Acidophilic fibrillar cytoplasm was detected in some acinar cells (Figure 1e). Pyknotic nuclei with ballooning and vacuolation of the cytoplasm of α and β cells were also evident (Figure 1f). In group IV (VPA and ALA treated group) the acini, exocrine, and endocrine compartments of the pancreas appeared as control groups apart from vacuolation of the cytoplasm of some acinar cells (Figure 1g–i).

**Figure 2:**
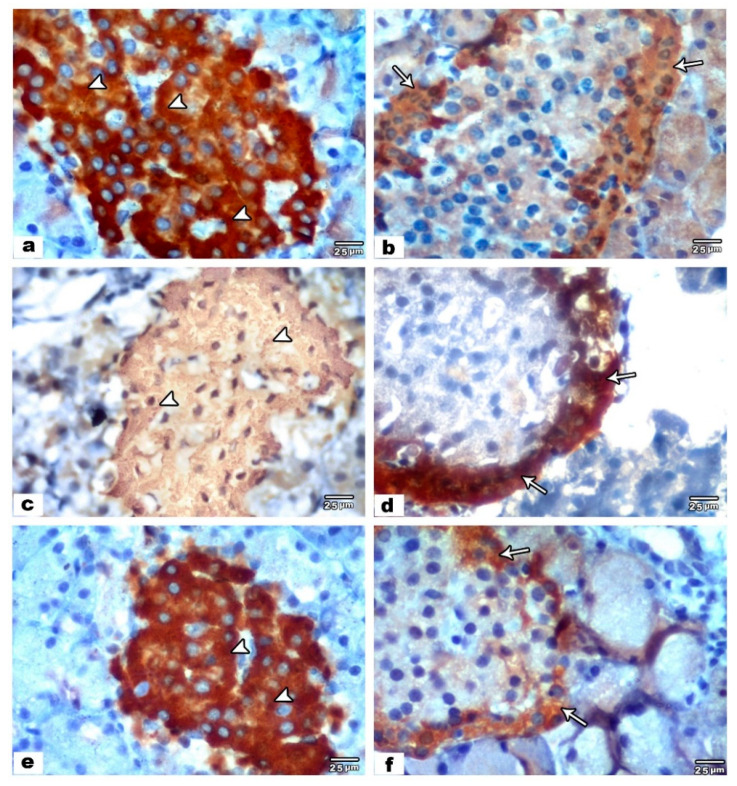
Photomicrographs of immunohistochemically stained pancreatic sections. Control sections (**a**,**b**). (**a**) An anti-insulin immune-reactivity of pancreatic islet showing brown-stained pancreatic β cells with strong positive immune-reaction (arrowheads). (**b**) An anti-glucagon immune-reactivity of pancreatic islet showing brown-stained pancreatic α cells with mild positive immune-reaction (arrow). VPA treated pancreatic sections (**c**,**d**). (**c**) An anti-insulin immune-reactivity of pancreatic islet showing brown-stained pancreatic β cells with mild positive immune-reaction (arrowheads). (**d**) An anti-glucagon immune-reactivity of pancreatic islet showing brown-stained pancreatic α cells with strong positive immune-reaction (arrow). VPA and ALA treated pancreatic sections (**e**,**f**). (**e**) An anti-insulin immune-reactivity of pancreatic islet showing brown-stained pancreatic β cells with strong positive immune-reaction (arrowheads). (**f**) An anti-glucagon immune-reactivity of pancreatic islet showing brown-stained pancreatic α-cells with mild positive immune-reaction (arrow).

In the immunohistochemically stained sections of control groups, islets of Langerhans demonstrated a strong positive cytoplasmic reaction in the form of brown granules when stained with anti-insulin antibody (Figure 2a) and a mild reaction when stained with anti-glucagon antibody (Figure 2b). The endocrine compartments of the offspring pancreas of group III (VPA treated group), revealed a weak positive cytoplasmic reaction when stained with anti-insulin antibody (Figure 2c) and a strong positive response when stained with anti-glucagon antibody (Figure 2d). In group IV (VPA and ALA treated group), the immunohistochemically stained sections (anti-insulin and anti-glucagon) were similar to control groups (Figure 2e,f).

#### 3.1.2. Electron Microscopic Results

**Figure 3:**
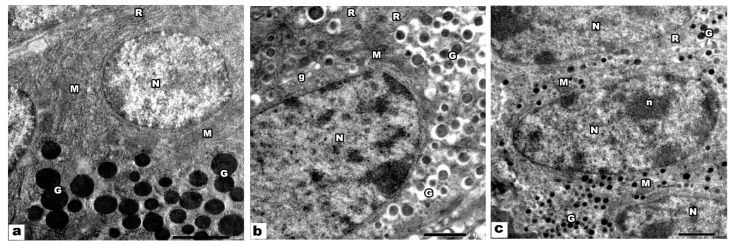
Electron micrograph of control pancreatic sections. (**a**) The acinar cells have basal, rounded, euchromatic nuclei (N), cytoplasm shows parallel cisternae of rough endoplasmic reticulum (R), mitochondria (M), and electron-dense granules (G). (**b**) β cells of the islets of Langerhans have euchromatic nuclei (N), and its cytoplasm is occupied by many electron-dense granules (G) encircled by clear space, mitochondria (M), rER (R), and golgi apparatus (g). (**c**) α cells have euchromatic nuclei (N) with prominent nucleoli (n), its cytoplasm is occupied by many electron-dense granules (G), rough endoplasmic reticulum (R), and mitochondria (M).

Electron microscopic examination of the pancreatic acini of control groups showed that the acinar cells had basal, rounded, euchromatic nuclei. The basal part of the cytoplasm was occupied by parallel cisternae of rough endoplasmic reticulum and mitochondria. In contrast, the apical region was occupied by electron-dense spherical secretory (zymogen) granules (Figure 3a). The endocrine compartments of the offspring pancreas of groups I and II was built chiefly of β cells that had euchromatic nuclei. Electron dense secretory granules encircled by a clear space, mitochondria, rER, and Golgi apparatus were detected in the cytoplasm of β cells (Figure 3b). α cells had euchromatic nuclei with prominent nucleoli, and its cytoplasm showed mitochondria, rER, and electron-dense secretory granules (Figure 3c).

**Figure 4:**
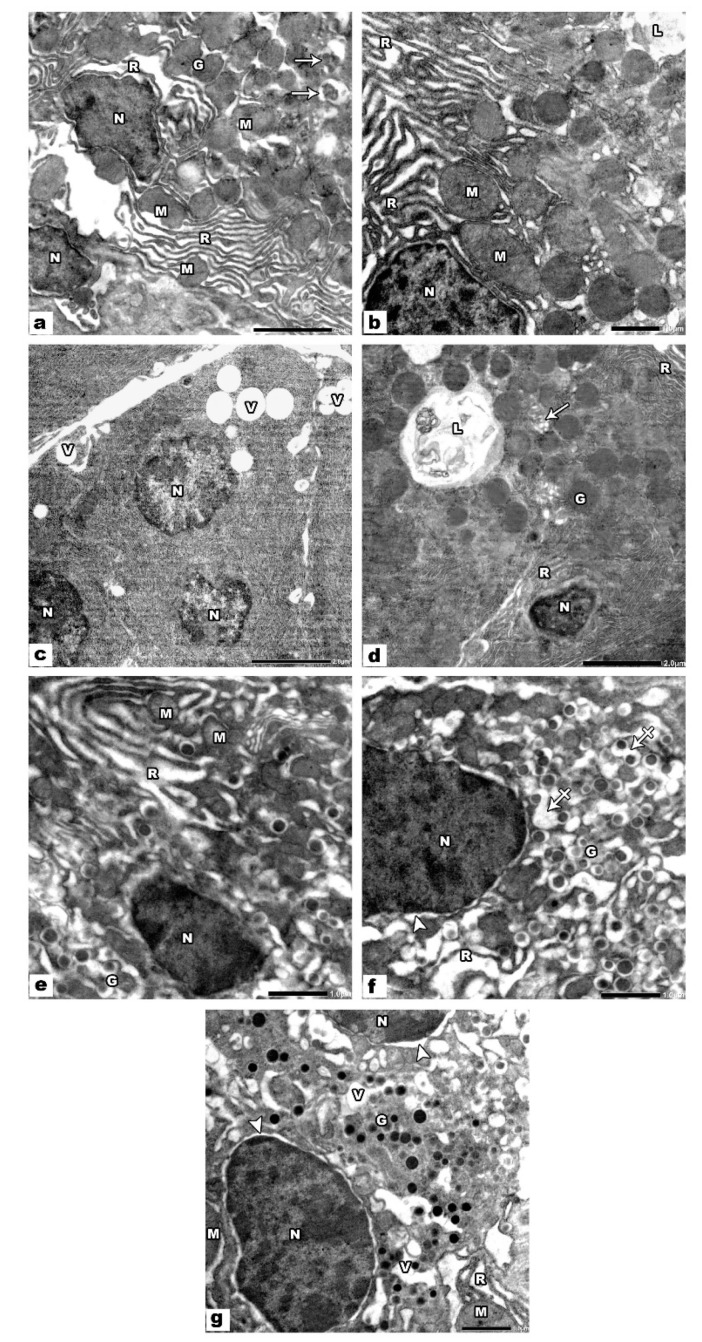
Electron micrographs of VPA treated pancreatic sections. (**a**–**d**) The pancreatic acini show small dense irregular heterochromatic nuclei (N), dilated disordered rough endoplasmic reticulum (R), swollen mitochondria (M), decreased secretory granules (G) some of them appear degraded (arrows), many cytoplasmic vacuoles (V) and autophagic vacuoles (L). (**e**,**f**) β cells of the islets show small dense irregular heterochromatic nuclei (N), some nuclei (N) show perinuclear halo (arrowhead), dilated rough endoplasmic reticulum (R), swollen mitochondria (M), increased lucent halo of many of the secretory granules (G) with the fusion of some of them (crossed arrows). (**g**) α cells of the islets show small irregular condensed nuclei (N) with wide perinuclear space (arrowhead), vacuolated cytoplasm (V), dilated disordered rER (R), swollen mitochondria (M), and secretory granules (G).

Ultrastructural analysis of the offspring pancreas of group III (VPA treated group) revealed noticeable changes in the acini, α cells, and β cells. Pancreatic acini appeared with small dense irregular heterochromatic nuclei, dilated disordered rough endoplasmic reticulum, and swollen mitochondria. The apparent decrease in the secretory granules was noticed in some acini, and some granules appeared degraded. Additionally, there were cytoplasmic vacuoles and autophagic vacuoles (Figure 4a–d). Regarding β cells of the islets of this group, they appeared with small dense irregular heterochromatic nuclei, and some nuclei appeared with perinuclear halos. Moreover, there were dilated rough endoplasmic reticulum, swollen mitochondria, and increased clear space of many of the secretory granules with a fusion of some granules (Figure 4e,f). α cells of the islets of this group exhibited small irregular condensed nuclei with wide perinuclear space, vacuolated cytoplasm, dilated disordered rough endoplasmic reticulum, and swollen mitochondria (Figure 4g).

**Figure 5:**
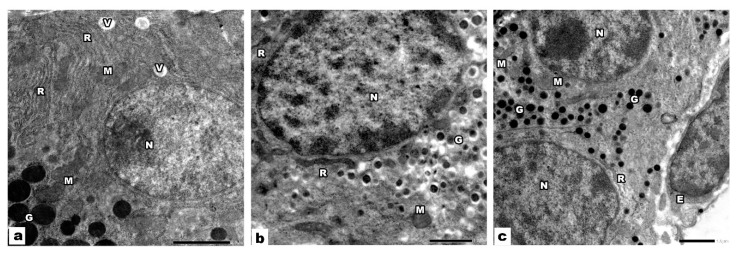
Electron micrographs of VPA and ALA treated pancreatic sections. (**a**) The acinar cells have rounded euchromatic nuclei (N), the cytoplasm shows parallel cisternae of rER (R), mitochondria (M), electron-dense granules (G), and small vacuoles (V). (**b**) β cells of the islets of Langerhans have euchromatic nuclei (N), electron-dense granules encircled by halo (G), mitochondria (M), and rER (R). (**c**) α cells have euchromatic nuclei (N), many electron-dense granules (G), rER (R), and mitochondria (M). Note the endothelial cell (E) of the blood capillary.

In group IV (VPA and ALA treated group), the pancreatic acini and islets of Langerhans appeared nearly as control groups except for the presence of small cytoplasmic vacuoles (Figure 5a–c).

### 3.2. Immunohistochemical and Morphometric Results

In groups I and II (control groups), there were non-significant changes in all morphometric parameters. In group III (VPA exposed rats), the percentage of insulin-positive cells was significantly decreased, and the percentage of glucagon positive cells was significantly increased in comparison to control groups (*p* < 0.001). In group IV (VPA and ALA treated rats), there was a significant increase in the percentage of insulin-positive cells and a significant decrease in the percentage glucagon positive cells in comparison to VPA exposed rats (*p* < 0.001). Non-significant changes in the percentage of both insulin and glucagon positive cells were detected in group IV compared to groups I and II (Table 1).

### 3.3. Biochemical and Molecular Results

In the control groups, a non-significant alteration in the levels of MDA, TAC, GSH, catalase activity, and SOD activity was observed in ALA treated rats (positive controls) when compared to the negative controls (*p* = 0.34, 0.85, 0.27, 0.37, and 0.45, respectively). In comparison to the control groups, a statistically significant increase in the level of MDA and a decrease in the levels of TAC, GSH, catalase activity, and SOD activity in VPA exposed rats (*p* < 0.001) was observed. Co-administration of ALA and VPA results in a significant decrease in MDA level with an increase in the levels of TAC, GSH, catalase activity, and SOD activity compared to VPA treated rats (*p* < 0.001) (Table 2).

These observed results in biochemical oxidative stress condition were supported by the molecular study as Nrf2 mRNA expression was significantly lower in VPA treated rat offspring pancreatic tissue samples (0.53 ± 0.08, *p* ˂ 0.001) and increased with co-administration of ALA and VPA (0.85 ± 0.13), however did not return to its normal level (Table 2). Moreover, alterations in gene expression of apoptosis-related genes (caspase-3 and Bcl-2) is in line with the histological results in pancreatic samples. VPA affects cells by stimulating apoptosis, where apoptotic caspase-3 gene expression was increased (2.8 ± 0.31), and anti-apoptotic BCL-2 expression decreased (0.32 ± 0.07). In contrast, ALA gave potential protection against apoptosis as it reversed the effect of VPA on caspase-3 and Bcl-2 gene expression (Table 2).

## 4. Discussion

Our study aimed to investigate the effect of prenatal administration of valproic acid on the pancreas of the rat offspring at the histological, immunohistochemical, ultrastructural, biochemical, and molecular levels. Moreover, it aimed to evaluate the potential protective role of prenatal administration of α-lipoic acid against VPA-induced pancreatic changes.

The use of harmless antiepileptic drugs (AEDs) during pregnancy is mandatory for two major purposes: to obtain a seizure-free period during pregnancy for the mother as well as to avoid teratogenic side effects of AEDs in the baby. There is a debate about the use of valproic acid (VPA) during pregnancy. Despite its possible teratogenic effect, it is prescribed for pregnant women with epilepsy, as reported by Macfarlane and Greenhalgh [3].

The pancreas is one of the organs most affected when the embryos are exposed to VPA, particularly throughout organ development [27]. At the developmental level, it could be explained as follows: prenatal administration of VPA during the organogenesis stage of pregnancy affects the gene expression of Pdx1, Nkx6.1, and Ngn3 genes [28]. Pdx1 has a significant role in the initial steps of the development of the pancreas [29]. Nkx6.1 mediates the branching of the pancreatic tip (which will form the exocrine compartment) and trunk segment (which will form the endocrine compartment) [8,30,31]. Ngn3 shares in the development of the endocrine part of the pancreas [32], its differentiation and insulin production [33]. Each of these genes are affected by VPA administration during embryogenesis.

Generally, the teratogenic effect of VPA could be explained by the ability of VPA to form a protein-bound VPA complex that can reach the embryo through the placenta in both humans and animals [34,35]. Exposure of the embryos to VPA coincides with the development of the targeted organs, including the pancreas [36]. Genetic as well as environmental factors shape the potential for VPA-induced pancreatic damage [37]. Inhibition of organogenesis and uncontrolled cell proliferation is explained by the ability of VPA to interfere with cell cycle processes, induce DNA damage, and cause epigenetic dysregulation via inhibition of histone deacetylase (HDAC) activity [38,39]. VPA-induced DNA damage leads to the instability of RNA synthesis and, therefore, protein synthesis [40]. VPA-induced endoplasmic reticulum stress is an additional mechanism of damage [41].

An important finding of the present study is that VPA-treated rats displayed higher markers of oxidative stress compared to the controls. This was characterized by an increased MDA level, diminished total antioxidant capacity (TAC), reduced expression of antioxidant enzyme activity (catalase and superoxide dismutase), and a lowered level of reduced glutathione. These biochemical findings were supported by decreased expression of the Nrf2 gene, which encodes a transcriptional factor regulating cellular antioxidant mechanisms, within pancreatic tissue samples from the VPA-treated rat offspring.

VPA-induced oxidative stress was previously documented by [41,42,43,44,45]. VPA stimulates intracellular reactive oxygen species (ROS) with the activation of redox-sensitive transcription factors that inhibit antioxidant response element-driven gene expression [14,43]. This oxidative stress was reported not only in animal studies but also in human studies [46].

Decreased Nrf2 gene expression is the proposed mechanism for VPA-induced oxidative stress [41,43,47]. Nrf2 is the transcription factor NF-E2-related factor 2. It enhances antioxidant production in response to the state of oxidative stress through a mechanism called the Nrf2/ARE pathway. Nrf2 binds to the antioxidant response element (ARE) that initiates expression of antioxidant genes (superoxide dismutase, glutathione reductase, and catalase) [48]. Moreover, it regulates the synthesis and maintenance of the normal level of reduced glutathione [49]. These molecular mechanisms could explain the relationship between the decreased biochemical level of these antioxidant enzymes and the decreased expression of Nrf2 in the current study.

The suppressor effect of VPA on Nrf2 gene expression in the current study is explained as follows: Nrf2 expression levels are directly sensitive to histone deacetylase (HDAC) enzymatic activity as histone deacetylase inhibitors induce hyperacetylation of chromatin proteins and, consequently, alter the gene expression of Nrf2 [49]. As a class I/II histone deacetylase inhibitor, VPA modulates Nrf2/ARE signaling pathway-regulated oxidative stress mechanisms through the inhibition of HDAC enzymatic activity [47]. This inhibition culminates in decreased Nrf2 gene expression and decreased Nrf2-dependent antioxidant capacity, as found in our study. In the current study, the decreased caspase-3 gene expression with an increase in Bcl-2 gene expression proved the previously documented role of VPA as an inducer of the intrinsic apoptotic pathway [50]. Both are linked to the mechanism involved in decreased expression of the Nrf2 gene [51].

This study revealed a decrease in the quantity of insulin-positive cells and an increase in the quantity of glucagon positive cells in the pancreas of VPA-treated rats. These results are in agreement with Komariah et al. [8]. They explained their findings by the effects of VPA on the production and development of insulin-producing β cells as well as glucagon-producing α cells in pups of VPA-treated mothers. The developmental disorders of both β and α cells are associated with disturbed function caused by a decrease in protein synthesis as a result of decreased RNA concentrations [52].

Examination of the pancreas by light microscope in our study revealed that VPA treatment led to vacuolated and eosinophilic fibrillar cytoplasm in some acinar cells. These findings coincide with that reported by [53]. They stated that vacuolization and eosinophilic fibrillar patterns in the acinar cell cytoplasm might be potential factors for cellular digestion. Additionally, there was ballooning of cells and pyknotic nuclei. Vacuolar degenerative changes and pyknotic nuclei were observed in the liver of mice treated with valproic acid [54].

Regarding α and β cells, we observed pyknotic nuclei with ballooning and vacuolation of the cytoplasm following VPA treatment. These endocrine cellular effects are partially explained by VPA-induced oxidative stress damage and partly due to VPA-dysregulated expression of Pdx1, Nkx6.1, and Ngn3 genes as mentioned before [8,28,29,31,32,33]. Oxidative stress could explain the light and electron microscopic changes of beta and alpha cells. These cells are very sensitive to ROS-related signaling and, after that, susceptible to oxidative and cytotoxicity stress due to the minimal antioxidant activity of the islets. This renders them in particular at risk for ROS-induced structural and functional damage [28].

On electron microscopic examination of the present work, the pancreatic acini showed small dense irregular heterochromatic nuclei, dilated disordered rER, swollen mitochondria, an apparent decrease in the secretory granules in some acini, and many cytoplasmic vacuoles and autophagy vacuoles. Similar findings were reported in another study on oxidative stress-induced pancreatic changes [55]. They studied the effect of sodium fluoride-triggered oxidative stress on the pancreas in the adult male albino rat, and found shrunken nuclei, dilated rER, cytoplasmic vacuoles, autophagosomes, and diminished zymogen granules. Stimulated autophagy of pancreatic acinar cells in response to VPA administration is considered as a defense mechanism by which the body tries to maintain pancreatic acinar cell homeostasis, normal protein synthesis activity for proper secretion of its secretions, and to prevent the occurrence of ER stress [56]. The link between VPA, oxidative stress, and enhanced autophagy is reported by Chen et al. [47] and was confirmed in our study at the ultrastructure level. β cells of the islets showed small, dense, irregular heterochromatic nuclei, with expanded (and, occasionally, fused) lucent halos of many of the secretory granules. α cells exhibited small, irregular, condensed nuclei with wide perinuclear space, vacuolated cytoplasm, dilated rough endoplasmic reticulum, and distorted mitochondria. Abdul-Hamid and Moustafa observed similar oxidative stress effects [57].

Alpha-lipoic acid (ALA) is a sulfur-containing organic compound produced by plants, animals, and humans. It acts as a cofactor for some enzymatic complexes involved in cellular energy production. It has promising therapeutic potential [58].

ALA is a naturally occurring antioxidant molecule. It improves and restores the intrinsic antioxidant systems, and stimulates endogenous antioxidant production [59]. It can sequester and remove oxidative stress-inducing heavy metals from the bloodstream. ALA can chelate ionic metals and neutralize their oxidizing effects, which gives it immense antioxidant capacity [60,61]. In addition to its antioxidant effect, ALA has an anti-inflammatory effect. A marked decrease in inflammatory markers (INF-γ, TGF-β, ICAM-1, and IL-4) was observed in ALA-treated animals in a study published by Khalili et al. [62]. Moreover, ALA has anti-apoptotic activity as proved by decreased caspase-3 and enhanced Bcl-2 gene expression in ALA-treated groups in the present study [63]. The cytoprotective roles of ALA were recently confirmed by Grandi et al., Castro et al., and Salehi et al. [58,64,65].

Improvement of oxidative stress parameters in the ALA-treated group of rats that receive VPA validates its role in counteracting VPA-induced oxidative stress. This improvement includes lowered MDA and increased TAC with increased levels of catalase and superoxide dismutase activity, and a higher level of reduced glutathione than the VPA-treated group. There is also increased Nrf2 gene expression in the pancreatic tissue of the offspring of the ALA treated rats. This finding coincides with that reported by Xia et al. [66]. The anti-apoptotic mechanism of ALA that is observed in our study (specifically, decreased caspase-3 with coordinated increased Bcl-2 genes expression) is reported by Marsh et al. and Park et al. [67,68].

Moreover, histological examination of the pancreas of offspring of VPA and ALA treated rats showed marked improvement at the light and electron microscopic levels. Immunoreactivity of ß cells and α cells also gave pictures nearly similar to the controls.

The cellular protective effect of ALA is in agreement with Go and Jones, whom propose that the role of ALA in modulating cell proliferation and inhibition of apoptosis antagonizes VPA action [69]. Moreover, ALA increases intracellular reduced glutathione levels and provides redox regulation of proteins and transcription factors that may have a stimulatory role in cell cycle progression [70]. The protective role of ALA on insulin-secreting cells was also documented in an animal study by Bruin et al. [71] and a human study by Cappellani et al. [72]. Decreased immunoreactivity of glucagon producing cells was previously confirmed by Topsakal et al. [63].

Co-administration of ALA and VPA in the current study was previously advised by Kulakli in his study with non-reported side effects and minimal drug–drug interaction between ALA and VA in microsomal metabolism and protein binding [73].

## 5. Conclusions

Prenatal valproic acid administration induces pancreatic oxidative stress, followed by cellular changes in the exocrine and endocrine compartments of the pancreas. These changes may affect the pancreatic function in the offspring of valproic acid-treated mothers, as proved by immunohistochemical examination of alpha and beta cells through a mechanism that involved oxidative stress and induction of apoptosis. α-lipoic acid co-administration with VPA significantly improved the oxidative stress condition and the histological and morphometric picture of the pancreas, and restored normal expression of some related genes, including Nrf2, caspase-3, and Bcl-2. Thus, the administration of the cytoprotective antioxidant α-lipoic acid might have a protective effect against VPA-induced pancreatic damage.

## Figures and Tables

**Table 1:** Insulin and glucagon positive cells (mean ± SD) of control and experimental groups.

	Group I (-ve Control)	Group II (+ve Control, ALA)	Group III (VPA Treated)	Group IV (VPA + ALA Treated)	ANOVA *p* Value	Tukey’s Test *p* Value
**Insulin positive cells (mean ± SD)**	83.6 ± 7.7	85.1 ± 2.1	57.3 ± 7.3	78.1 ± 4.6	˂0.001 *	P1 = 1.00
						P2 ˂ 0.001 *
						P3 = 0.37
						P4 ˂ 0.001 *
**Glucagon positive cells (mean ± SD)**	29.9 ± 4.5	30.8 ± 6.9	41.2 ± 4.9	26.6 ± 7.5	˂0.001 *	P1 = 0.99
						P2 ˂ 0.001 *
						P3 = 0.79
						P4 ˂ 0.001 *

P1: ALA treated group (II) versus control group (I), P2: VPA treated group (III) versus control group (I), P3: VPA plus ALA treated group (IV) versus control group (I), P4: VPA treated group (III) versus VPA plus ALA treated group (IV), * means significant.

**Table 2:** The biochemical and molecular parameters (mean ± SD) of control and experimental groups.

	Group I (-ve Control)	Group II (+ve Control, ALA)	Group III (VPA Treated)	Group IV (VPA + ALA Treated)	ANOVA *p* Value	Tukey’s Test *p* Value
MDA (nmol/g tissue)	9.49 ± 1.04	7.78 ± 0.33	23.87 ± 3.29	10.47 ± 0.54	˂0.001 *	P1 = 0.34, P2 ˂ 0.001 *, P3 = 0.76, P4 ˂ 0.001 *
TAC (mmol/L)	978.5 ± 33.3	996.8 ± 24.2	355.9 ± 33.9	1030.3 ± 54.5	˂0.001 *	P1 = 0.85, P2 ˂ 0.001 *, P3 = 0.12, P4 ˂ 0.001 *
GSH (μmol/mL)	156.2 ± 20.2	174.1 ± 20.2	50.7 ± 12.2	169.4 ± 11.5	˂0.001 *	P1 = 0.27, P2 ˂ 0.001 *, P3 = 0.96, P4 ˂ 0.001 *
Catalase (U/mL)	552.6 ± 46.9	595.1 ± 55.6	403.2 ± 43.7	545.1 ± 25.4	˂0.001 *	P1 = 0.37, P2 ˂ 0.001 *, P3 = 0.24, P4 ˂ 0.001 *
SOD (U/mL)	86.8 ± 7.2	94.1 ± 5.3	68.4 ± 16.7	80.4 ± 10.7	˂0.001 *	P1 = 0.45, P2 ˂ 0.001 *, P3 = 0.55, P4 ˂ 0.001 *
Nrf2 gene expression (2^−∆∆CT^) Mean ± SD	1.00 ± 0.10	1.00 ± 0.11	0.53 ± 0.08	0.85 ± 0.13	˂0.001 *	P1 = 0.36, P2 ˂ 0.001 *, P3 = 0.16, P4 ˂ 0.001 *
Caspase-3 gene expression (2^−∆∆CT^) Mean ± SD	1.00 ± 0.09	1.1 ± 0.21	2.8 ± 0.31	2.3 ± 0.16	˂0.001 *	P1 = 0.54, P2 ˂ 0.001 *, P3 = 0.05*, P4 ˂ 0.001 *
Bcl-2 gene expression (2^−∆∆CT^) Mean ± SD	1.00 ± 0.10	1.08 ± 0.24	0.32 ± 0.07	0.87 ± 0.11	˂0.001 *	P1 = 0.43, P2 ˂ 0.001 *, P3 = 0.21, P4 ˂ 0.001 *

P1: ALA treated group (II) versus control group (I), P2: VPA treated group (III) versus control group (I), P3: VPA plus ALA treated group (IV) versus control group (I), P4: VPA treated group (III) versus VPA plus ALA treated group (IV), * means significant.

## References

[1] Spina E., Perugi G. (2004). Antiepileptic drugs: Indications other than epilepsy. Epileptic Disord..

[2] Veroniki A.A., Rios P., Cogo E., Straus S.E., Finkelstein Y., Kealey R., Reynen E., Soobiah C., Thavorn K., Hutton B. (2017). Comparative safety of antiepileptic drugs for neurological development in children exposed during pregnancy and breast feeding: A systematic review and network meta-analysis. BMJ Open.

[3] Macfarlane A., Greenhalgh T. (2018). Sodium valproate in pregnancy: What are the risks and should we use a shared decision-making approach?. BMC Pregnancy Childbirth.

[4] Wieck A., Jones S. (2018). Dangers of valproate in pregnancy. BMJ.

[5] Harden C.L., Pennell P.B., Koppel B.S., Hovinga C.A., Gidal B., Meador K.J., Hopp J., Ting T.Y., Hauser W.A., Thurman D. (2009). Management issues for women with epilepsy-Focus on pregnancy (an evidence-based review): III. Vitamin K, folWic acid, blood levels, and breast-feeding. Epilepsia.

[6] Petersen I., Collings S.L., McCrea R.L., Nazareth I., Osborn D.P., Cowen P.J., Sammon C.J. (2017). Antiepileptic drugs prescribed in pregnancy and prevalence of major congenital malformations: Comparative prevalence studies. Clin. Epidemiol..

[7] da Costa R.F.M., Kormann M.L., Galina A., Rehen S.K. (2015). Valproate Disturbs Morphology and Mitochondrial Membrane Potential in Human Neural Cells. Appl. In Vitro Toxicol..

[8] Komariah K., Manalu W., Kiranadi B., Winarto A., Handharyani E., Roeslan M.O. (2018). Valproic Acid Exposure of Pregnant Rats During Organogenesis Disturbs Pancreas Development in Insulin Synthesis and Secretion of the Offspring. Toxicol. Res..

[9] Hamzawy M.A., El-Ghandour Y.B., Abdel-Aziem S.H., Ali Z.H. (2018). Leptin and camel milk abate oxidative stress status, genotoxicity induced in valproic acid rat model of autism. Int. J. Immunopathol. Pharmacol..

[10] Jain A., Haque I., Tayal V., Roy V. (2019). Valproic acid-induced acute pancreatitis. Indian J. Psychiatry.

[11] Ma X.J., Wang Y.S., Gu W.P., Zhao X. (2017). The role and possible molecular mechanism of valproic acid in the growth of MCF-7 breast cancer cells. Croat. Med. J..

[12] Badran M., Abuyassin B., Golbidi S., Ayas N., Laher I. (2019). Alpha Lipoic Acid Improves Endothelial Function and Oxidative Stress in Mice Exposed to Chronic Intermittent Hypoxia. Oxid. Med. Cell. Longev..

[13] Tian Y.F., He C.T., Chen Y.T., Hsieh P.S. (2013). Lipoic acid suppresses portal endotoxemia-induced steatohepatitis and pancreatic inflammation in rats. World J. Gastroenterol..

[14] Parente E., Colannino G., Picconi O., Monastra G. (2017). Safety of oral alpha-lipoic acid treatment in pregnant women: A retrospective observational study. Eur. Rev. Med. Pharmacol..

[15] Al-Matubsi H.Y., Oriquat G.A., Abu-Samak M., al Hanbali O.A., Salim M.D. (2017). Corrigendum to “Effects of Lipoic Acid Supplementation on Activities of Cyclooxygenases and Levels of Prostaglandins E2 and F2alpha Metabolites, in the Offspring of Rats with Streptozotocin-Induced Diabetes”. J. Diabetes Res..

[16] Schneider T., Turczak J., Przewlocki R. (2006). Environmental enrichment reverses behavioral alterations in rats prenatally exposed to valproic acid: Issues for a therapeutic approach in autism. Neuropsychopharmacol. Off. Publ. Am. Coll. Neuropsychopharmacol..

[17] Bancroft J.D., Layton C., Suvarna S.K., Layton C., Bancroft J.D. (2019). 10—The hematoxylins and eosin. Bancroft’s Theory and Practice of Histological Techniques.

[18] Sciau P., Hawkes P.W. (2016). Chapter Two—Transmission Electron Microscopy: Emerging Investigations for Cultural Heritage Materials. Advances in Imaging and Electron Physics.

[19] Cemek M., Kaga S., Simsek N., Buyukokuroglu M.E., Konuk M. (2008). Antihyperglycemic and antioxidative potential of Matricaria chamomilla L. in streptozotocin-induced diabetic rats. J. Nat. Med..

[20] Draper H.H., Squires E.J., Mahmoodi H., Wu J., Agarwal S., Hadley M. (1993). A comparative evaluation of thiobarbituric acid methods for the determination of malondialdehyde in biological materials. Free Radic. Biol. Med..

[21] Koracevic D., Koracevic G., Djordjevic V., Andrejevic S., Cosic V. (2001). Method for the measurement of antioxidant activity in human fluids. J. Clin. Pathol..

[22] Koroliuk M.A., Ivanova L.I., Maiorova I.G., Tokarev V.E. (1988). A method of determining catalase activity. Lab. Delo.

[23] Wendel A. (1981). Glutathione peroxidase. Methods in Enzymology.

[24] Sun Y., Oberley L.W., Li Y. (1988). A Simple Method for Clinical Assay of Superoxide-Dismutase. Clin. Chem..

[25] Yamashita Y., Ueyama T., Nishi T., Yamamoto Y., Kawakoshi A., Sunami S., Iguchi M., Tamai H., Ueda K., Ito T. (2014). Nrf2-inducing anti-oxidation stress response in the rat liver—New beneficial effect of lansoprazole. PLoS ONE.

[26] He X., Sun J., Huang X. (2018). Expression of caspase-3, Bax and Bcl-2 in hippocampus of rats with diabetes and subarachnoid hemorrhage. Exp. Ther. Med..

[27] Ornoy A., Ergaz Z. (2010). Alcohol Abuse in Pregnant Women: Effects on the Fetus and Newborn, Mode of Action and Maternal Treatment. Int. J. Environ. Res. Public Health.

[28] Wang J., Wang H. (2017). Oxidative Stress in Pancreatic Beta Cell Regeneration. Oxid. Med. Cell. Longev..

[29] Otsuka T., Tsukahara T., Takeda H. (2015). Development of the pancreas in medaka, Oryzias latipes, from embryo to adult. Dev. Growth Differ..

[30] Wang X., Wei X., Pang Q., Yi F. (2012). Histone deacetylases and their inhibitors: Molecular mechanisms and therapeutic implications in diabetes mellitus. Acta Pharm. Sin. B.

[31] Sostrup B., Gaarn L.W., Nalla A., Billestrup N., Nielsen J.H. (2014). Co-ordinated regulation of neurogenin-3 expression in the maternal and fetal pancreas during pregnancy. Acta Obstet. Gyn. Scan.

[32] Gomez D.L., O’Driscoll M., Sheets T.P., Hruban R.H., Oberholzer J., McGarrigle J.J., Shamblott M.J. (2015). Neurogenin 3 Expressing Cells in the Human Exocrine Pancreas Have the Capacity for Endocrine Cell Fate. PLoS ONE.

[33] Martinez-Sanchez A., Rutter G.A., Latreille M. (2016). MiRNAs in beta-Cell Development, Identity, and Disease. Front. Genet..

[34] Guerrini R. (2006). Valproate as a Mainstay of Therapy for Pediatric Epilepsy. Pediatr. Drugs.

[35] De Felice A., Ricceri L., Venerosi A., Chiarotti F., Calamandrei G. (2015). Multifactorial Origin of Neurodevelopmental Disorders: Approaches to Understanding Complex Etiologies. Toxics.

[36] Kokate P., Bang R. (2017). Study of congenital malformation in tertiary care centre, Mumbai, Maharashtra, India. Int. J. Reprod. Contracept. Obstet. Gynecol..

[37] Wlodarczyk B.J., Palacios A.M., Chapa C.J., Zhu H., George T.M., Finnell R.H. (2011). Genetic basis of susceptibility to teratogen induced birth defects. Am. J. Med. Genet. Part C Semin. Med. Genet..

[38] Bertoli C., Skotheim J.M., de Bruin R.A. (2013). Control of cell cycle transcription during G1 and S phases. Nat. Rev. Mol. Cell Biol..

[39] Li Q., Foote M., Chen J. (2014). Effects of histone deacetylase inhibitor valproic acid on skeletal myocyte development. Sci. Rep..

[40] Shkreta L., Chabot B. (2015). The RNA Splicing Response to DNA Damage. Biomolecules.

[41] Palsamy P., Bidasee K.R., Shinohara T. (2014). Valproic acid suppresses Nrf2/Keap1 dependent antioxidant protection through induction of endoplasmic reticulum stress and Keap1 promoter DNA demethylation in human lens epithelial cells. Exp. Eye Res..

[42] Defoort E.N., Kim P.M., Winn L.M. (2006). Valproic acid increases conservative homologous recombination frequency and reactive oxygen species formation: A potential mechanism for valproic acid-induced neural tube defects. Mol. Pharmacol..

[43] Kawai Y., Arinze I.J. (2006). Valproic acid-induced gene expression through production of reactive oxygen species. Cancer Res..

[44] Arafat E.A., Shabaan D.A. (2019). The possible neuroprotective role of grape seed extract on the histopathological changes of the cerebellar cortex of rats prenatally exposed to Valproic Acid: Animal model of autism. Acta Histochem..

[45] Ornoy A., Weinstein-Fudim L., Ergaz Z. (2019). Prevention or Amelioration of Autism-Like Symptoms in Animal Models: Will it Bring Us Closer to Treating Human ASD?. Int. J. Mol. Sci..

[46] Beltran-Sarmiento E., Arregoitia-Sarabia C.K., Floriano-Sanchez E., Sandoval-Pacheco R., Galvan-Hernandez D.E., Coballase-Urrutia E., Carmona-Aparicio L., Ramos-Reyna E., Rodriguez-Silverio J., Cardenas-Rodriguez N. (2018). Effects of Valproate Monotherapy on the Oxidant-Antioxidant Status in Mexican Epileptic Children: A Longitudinal Study. Oxid. Med. Cell. Longev..

[47] Chen X., Wang H., Zhou M., Li X., Fang Z., Gao H., Li Y., Hu W. (2018). Valproic Acid Attenuates Traumatic Brain Injury-Induced Inflammation in Vivo: Involvement of Autophagy and the Nrf2/ARE Signaling Pathway. Front. Mol. Neurosci..

[48] Wasik U., Milkiewicz M., Kempinska-Podhorodecka A., Milkiewicz P. (2017). Protection against oxidative stress mediated by the Nrf2/Keap1 axis is impaired in Primary Biliary Cholangitis. Sci. Rep..

[49] Zhang X.S., Wu Q., Wu L.Y., Ye Z.N., Jiang T.W., Li W., Zhuang Z., Zhou M.L., Zhang X., Hang C.H. (2016). Sirtuin 1 activation protects against early brain injury after experimental subarachnoid hemorrhage in rats. Cell Death Dis..

[50] Aalaei S., Mohammadzadeh M., Pazhang Y. (2019). Synergistic induction of apoptosis in a cell model of human leukemia K562 by nitroglycerine and valproic acid. EXCLI J..

[51] Hassanein E.H.M., Shalkami A.-G.S., Khalaf M.M., Mohamed W.R., Hemeida R.A.M. (2019). The impact of Keap1/Nrf2, P38MAPK/NF-κB and Bax/Bcl2/caspase-3 signaling pathways in the protective effects of berberine against methotrexate-induced nephrotoxicity. Biomed. Pharmacother..

[52] Olivar M.P., Diaz M.V., Chicharo M.A. (2009). Tissue effect on RNA:DNA ratios of marine fish larvae. Sci. Mar..

[53] Oktay S., Alev-Tuzuner B., Tunali S., Ak E., Emekli-Alturfan E., Tunali-Akbay T., Koc-Ozturk L., Cetinel S., Yanardag R., Yarat A. (2017). Investigation of the Effects of Edaravone on Valproic Acid Induced Tissue Damage in Pancreas. Marmara Pharm. J..

[54] Al-Amoudi W.M. (2017). Protective effects of fennel oil extract against sodium valproate-induced hepatorenal damage in albino rats. Saudi J. Biol. Sci..

[55] Zaghloul D., Gad-El-Rab W.M., Bushra R.R., Farahat A.A. (2019). The Possible Protective Role of Methionine against Sodium Fluoride-Induced Pancreatic Changes in the Adult Male Albino Rat: A Histological, Immunohistochemical and Morphometric Study. Egypt. J. Histol..

[56] Antonucci L., Fagman J.B., Kim J.Y., Todoric J., Gukovsky I., Mackey M., Ellisman M.H., Karin M. (2015). Basal autophagy maintains pancreatic acinar cell homeostasis and protein synthesis and prevents ER stress. Proc. Natl. Acad. Sci. USA.

[57] Abdul-Hamid M., Moustafa N. (2013). Protective effect of curcumin on histopathology and ultrastructure of pancreas in the alloxan treated rats for induction of diabetes. J. Basic Appl. Zool..

[58] Salehi B., Berkay Yılmaz Y., Antika G., Boyunegmez Tumer T., Fawzi Mahomoodally M., Lobine D., Akram M., Riaz M., Capanoglu E., Sharopov F. (2019). Insights on the Use of α-Lipoic Acid for Therapeutic Purposes. Biomolecules.

[59] Gorąca A., Huk-Kolega H., Piechota A., Kleniewska P., Ciejka E., Skibska B. (2011). Lipoic acid–biological activity and therapeutic potential. Pharmacol. Rep..

[60] Shay K.P., Moreau R.F., Smith E.J., Smith A.R., Hagen T.M. (2009). Alpha-lipoic acid as a dietary supplement: Molecular mechanisms and therapeutic potential. BBA-Gen. Subj..

[61] Mendoza-Nunez V.M., Garcia-Martinez B.I., Rosado-Perez J., Santiago-Osorio E., Pedraza-Chavem J., Hernandez-Abad V.J. (2019). The Effect of 600 mg Alpha-lipoic Acid Supplementation on Oxidative Stress, Inflammation, and RAGE in Older Adults with Type 2 Diabetes Mellitus. Oxid. Med. Cell. Longev..

[62] Khalili M., Azimi A., Izadi V., Eghtesadi S., Mirshafiey A., Sahraian M.A., Motevalian A., Norouzi A., Sanoobar M., Eskandari G. (2014). Does Lipoic Acid Consumption Affect the Cytokine Profile in Multiple Sclerosis Patients: A Double-Blind, PlaceboControlled, Randomized Clinical Trial. Neuroimmunomodulation.

[63] Topsakal S., Ozmen O., Aslankoc R., Aydemir D.H. (2016). Pancreatic damage induced by cigarette smoke: The specific pathological effects of cigarette smoke in the rat model. Toxicol. Res. UK.

[64] Grandi G., Pignatti L., Ferrari F., Dante G., Neri I., Facchinetti F. (2017). Vaginal alpha-lipoic acid shows an anti-inflammatory effect on the cervix, preventing its shortening after primary tocolysis. A pilot, randomized, placebo-controlled study. J. Matern-Fetal Neonatal Med..

[65] Castro M.C., Villagarcia H.G., Massa M.L., Francini F. (2019). Alpha-lipoic acid and its protective role in fructose induced endocrine-metabolic disturbances. Food Funct..

[66] Xia D., Zhai X., Wang H., Chen Z., Fu C., Zhu M. (2019). Alpha lipoic acid inhibits oxidative stress-induced apoptosis by modulating of Nrf2 signalling pathway after traumatic brain injury. J. Cell. Mol. Med..

[67] Marsh S.A., Pat B.K., Gobe G.C., Coombes J.S. (2005). Evidence for a non-antioxidant, dose-dependent role of alpha-lipoic acid in caspase-3 and ERK2 activation in endothelial cells. Apoptosis.

[68] Park J.S., Choi H.I., Kim D.H., Kim C.S., Bae E.H., Ma S.K., Kim S.W. (2019). Alpha-lipoic acid attenuates p-cresyl sulfate-induced renal tubular injury through suppression of apoptosis and autophagy in human proximal tubular epithelial cells. Biomed. Pharmacother..

[69] Go Y.M., Jones D.P. (2010). Redox clamp model for study of extracellular thiols and disulfides in redox signaling. Methods Enzymol..

[70] Packer L., Witt E.H., Tritschler H.J. (1995). Alpha-Lipoic Acid as a Biological Antioxidant. Free Radic. Biol. Med..

[71] Bruin J.E., Woynillowicz A.K., Hettinga B.P., Tarnopolsky M.A., Morrison K.M., Gerstein H.C., Holloway A.C. (2012). Maternal antioxidants prevent beta-cell apoptosis and promote formation of dual hormone-expressing endocrine cells in male offspring following fetal and neonatal nicotine exposure. J. Diabetes.

[72] Cappellani D., Sardella C., Campopiano M.C., Falorni A., Marchetti P., Macchia E. (2018). Spontaneously remitting insulin autoimmune syndrome in a patient taking alpha-lipoic acid. Endocrinol. Diabetes Metab. Case Rep..

[73] Kulakli F. (2018). Effect of Alpha Lipoic Acid in the Treatment of Multiple Sclerosis-Induced Neuropathic Pain: A Case Report. Eurasian J. Med. Oncol..

